# Biological recognition of graphene nanoflakes

**DOI:** 10.1038/s41467-018-04009-x

**Published:** 2018-04-20

**Authors:** V. Castagnola, W. Zhao, L. Boselli, M. C. Lo Giudice, F. Meder, E. Polo, K. R. Paton, C. Backes, J. N. Coleman, K. A. Dawson

**Affiliations:** 1 Centre for BioNano Interactions, School of Chemistry, University College Dublin, Belfield, Dublin, 4 Ireland; 2 School of Physics, CRANN and AMBER, Trinity College Dublin, Dublin, 2 Ireland

## Abstract

The systematic study of nanoparticle–biological interactions requires particles to be reproducibly dispersed in relevant fluids along with further development in the identification of biologically relevant structural details at the materials–biology interface. Here, we develop a biocompatible long-term colloidally stable water dispersion of few-layered graphene nanoflakes in the biological exposure medium in which it will be studied. We also report the study of the orientation and functionality of key proteins of interest in the biolayer (corona) that are believed to mediate most of the early biological interactions. The evidence accumulated shows that graphene nanoflakes are rich in effective apolipoprotein A-I presentation, and we are able to map specific functional epitopes located in the C-terminal portion that are known to mediate the binding of high-density lipoprotein to binding sites in receptors that are abundant in the liver. This could suggest a way of connecting the materials' properties to the biological outcomes.

## Abstract (web summary)

The production of graphene nanoflakes for biological studies is usually done in a controlled chemical environment. Here, the authors develop a method to produce graphene nanoflakes in biofluids and find that their protein coats are different from that of other materials.

## Introduction

Recent years have seen very active interest in understanding the factors that influence nanoparticle interactions with living systems^1–3^. Among the other nanomaterials, the distinctive properties of graphene^4, 5^ have attracted an immense scientific interest and have raised high expectations for its potential applications not only in the field of electronics, photonics, composite materials, and energy generation and storage, but also in biological fields^6–9^.

In evaluating the interaction of graphene with cells^10–12^, one could expect distinctive interactions because of the distinctive shape, size^13–15^, surface functionalization^16–18^, as well as the surface presentations to cellular- processing machinery^19–21^. The question of nanosurface presentation is not unique to graphene, but the challenges here are more significant than for most systems. Thus, we now believe that in contact with biological media, the bare nanoparticle surface induces the formation of a relatively slowly exchanging layer of molecules derived from the environment, often modeled simply by animal serum or plasma. This has been named the “biomolecular corona”^22–24^. Increasingly, we recognize that besides shape, it is the biomolecular recognition motifs conferred by this layer which lead to most early-stage impacts (in vitro cell-interaction studies, immunological response, and biodistribution). Indeed, recent capacity to “read” or map out these aspects of the corona^25–27^ reinforces the role that the proteins play in most early stages of biological interactions^28^. The nature of the graphene surface makes these issues even more significant, as it is difficult for first-exposure (exfoliation) dispersants, once they have formed such an adsorbed layer to be displaced. In usual circumstances though, one may expect the (surface-induced) biological identity to be determined by the first dispersant (or exposure scenario) encountered. Thus, one has the choice between progressive dispersion of a more weakly interacting dispersant that is then subsequently displaced in exposure (and establishing that this displacement is indeed complete, and ensuring that the dispersant has no competing biological interactions) or creating dispersions directly in the exposure medium of interest^29^. It would in general be desirable to do both, and establish the nature of the receptor interactions that result^28,30^. Typically, it is also understood that the source of the media (sera, plasma, or other fluids) should be matched to the origin of cells in any in vitro study, as proteins and other biomolecules from different species may interact differently with receptors from another species. One should be aware that the organization of the recognition motifs on the nanoparticle surface may also be affected by the nature of the displacement or attachment, and in any case, it will be important to use biological media that reflect the real exposure scenario. Thus, lung fluids will be important for inhalation scenarios, and in situ exfoliation may be most appropriate^31^. These are all highly significant issues that touch on the whole validity and durability of such studies^32^.

In the present paper (using human and fetal calf serum as models), we show that it is possible to exfoliate directly in the presence of a biological milieu, without undergoing uncontrolled oxidation/reduction of functionalization processes (therefore affecting the graphene properties) and without compromising the protein functionality, thereby allowing for an interesting and useful model of biological exposure scenarios. We also show that it is possible to characterize the dispersed graphene, both in relation to size, and the number of layers in the nanoflakes. Finally, we isolate the graphene–corona complexes, analyzing the macroscopically averaged composition of the hard-corona layer. While some of the proteins identified are common to many other nanomaterials, some typical ones are absent (e.g., apolipoprotein B100), and some are unusually abundant (e.g., apolipoprotein A-I). These differences may be reflected in the short-term biological outcomes for graphene.

## Results

### Graphene nanoflakes protein corona composition

Graphene can be produced starting from graphite, by a large variety of techniques, even though reliable production of biologically dispersed single-layer graphene samples in high quality and yield is still a challenge^33^. Top-down approaches for graphene nanoflakes production have focused on the separation of graphite planes using, e.g., ultrasonic or shear exfoliation in organic solvents or water-based surfactant solutions^34–36^. Liquid-phase exfoliation (LPE) of graphite is a common method to obtain graphene water dispersions, and the use of biomolecules as a means to produce biocompatible graphene dispersions has recently attracted increasing attention^37–40^. A common choice has been to disperse graphene (and other carbon materials), making use of single proteins. However, because our strategy is to explore biological interactions, we employ the full-protein portfolio (complete serum) as a model for realistic biological exposure scenarios. This leads to presentation of appropriate biological recognition motifs.

In this work, highly stable colloidal dispersions of graphene nanoflakes in aqueous solutions were prepared by 1–4 h of bath sonication of natural flake graphite in complete serum and phosphate-buffered saline (PBS). Different kinds of serum and concentrations were investigated. This process, described in more detail in Methods, resulted in a mixture of graphite and graphene with different thicknesses and lateral sizes. The dispersions were therefore subjected to a size selection by centrifugation (Supplementary Fig. 1a), and unbound proteins in excess were removed by high-speed centrifugation. As an alternative to the size selection, different sonication times can also be used to tune the size distribution of the flakes (Supplementary Fig. 1d).

**Fig. 1:**
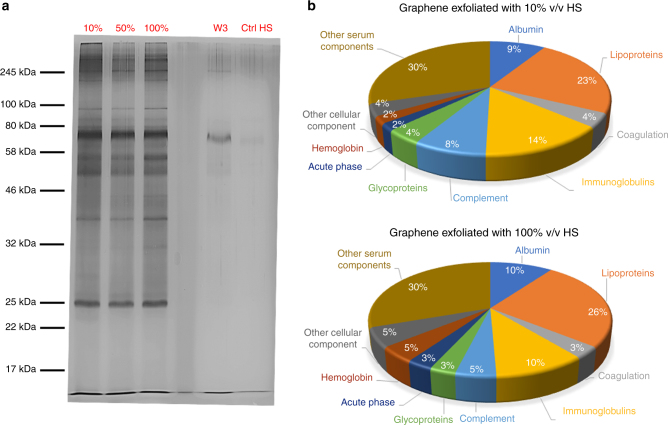
Proteomic analysis of graphene dispersions exfoliated with different concentrations of proteins. **a** 1D SDS-PAGE analysis of graphene dispersions exfoliated with 10, 50, and 100% v/v HS. Proteins were silver-stained. The controls represent both HS subjected to the same procedure (ultrasonication and centrifugation, Ctrl HS), considered as a background, and the supernatant collected after three washing steps (W3 ctrl). The protein profile is not modified above a certain protein concentration (50% v/v). **b** Pie chart indicating the relative coverage of protein, as identified by mass spectrometry onto the graphene flakes (exfoliated with 10% v/v and 100% v/v of HS) and organized per protein function. A clear predominance of albumin, lipoproteins, and immunoglobulins can be noticed

During this exfoliation process, a layer of protein adsorbs onto the graphene flakes, surrounding them and stabilizing the highly hydrophobic graphene surface in water. The composition and orientation of this protein layer, with a high affinity for the graphene surface, represent the final biological identity of the graphene nanoflakes, and the key biological motives presented at the periphery will be eventually interacting with the surface of cells. The protein composition was resolved by proteomic analysis. Here, we report the results obtained using human serum (HS) at different concentrations (Fig. 1a and Table 1), while the results related to the use of fetal bovine serum (FBS) can be found in Supplementary Fig. 2–4 and Supplementary Data 1 and 2.

**Table 1:** List of highly abundant proteins on the surface of graphene exfoliated in 100% human serum as identified by MS

Protein ID	Protein name	Mol. weight (kDa)	NSpC%
P02768	Serum albumin	69.366	10.02
P02647	Apolipoprotein A-I	30.777	8.55
P02649	Apolipoprotein E	36.154	4.40
P04004	Vitronectin	54.305	4.30
P01009	Alpha-1-antitrypsin	46.736	3.40
P06727	Apolipoprotein A-IV	45.398	3.17
P68871	Hemoglobin subunit beta	15.998	3.10
V9GYE3	Apolipoprotein A-II	5.8767	2.53
P01024	Complement C3	187.15	2.41
P01008	Antithrombin-III	52.602	2.08
P04196	Histidine-rich glycoprotein	59.578	1.92
P10909	Clusterin	48.803	1.63
P02656	Apolipoprotein C-III	10.852	1.37
P0CG06	Ig lambda	11.237	1.32
P69905	Hemoglobin subunit alpha	15.257	1.30

The proteins are ordered by relative abundance calculated by the method of normalized spectral counts (NSpC), as analyzed by MaxQuant 1.4.1.2.

The proteins surrounding the flakes were denatured (procedure described in the Methods section) and separated from graphene flakes using 1D sodium dodecyl sulfate polyacrylamide gel electrophoresis (SDS-PAGE), as reported in Fig. 1a. The concentrations of graphene dispersions of the same size distribution were normalized using the extinction values at 800 nm. At this wavelength, the extinction coefficient of graphene is size independent and also not influenced by the presence of proteins, as shown in Supplementary Fig. 5 and directly correlates to the graphene concentration^41^.

Figure 1a shows the protein profile for graphene nanoflakes exfoliated with different concentrations of HS. Two reference controls have been included in the gel to assure that (1) no proteins are left in the supernatants after the last washing step and that (2) no protein aggregates that might form during the sonication procedure are left in the graphene dispersion which would otherwise affect the protein analysis.

To better resolve the protein composition on the graphene nanoflakes, mass-spectrometry (MS) analysis was performed, as detailed in the Methods section. Three bioinformatics analyses were used for comparison purposes (more details in the Methods section). Table 1 presents a list of proteins highly abundant on the graphene nanoflakes surface when exfoliated with 100% v/v HS, as identified by MS and analyzed by MaxQuant. For better clarity, only the most abundant proteins (matched with the other two analyses described in the Methods section) are reported in Table 1. The complete list of proteins analyzed by MaxQuant as normalized spectral counts (NSpC) can be found in Supplementary Data 1 and 2.

The pie charts in Fig. 1b represent the statistical distribution of proteins (organized by class) on the graphene flakes when the concentration of serum is varied. From these charts and Table 1, the following considerations can be made: (i) human serum albumin (HSA) seemed to have a strong affinity for the graphene flakes under these exfoliation conditions, and this has also been found in the literature for graphene oxide (GO) and carbon nanotubes^42–46^; (ii) lipoproteins and immunoglobulins played a major role when HS was used for exfoliation, while, as shown in Supplementary Fig. 3, the presence of hemoglobin and opsonin proteins is remarkable when FBS is used; and (iii) the serum concentration seems to slightly affect the overall corona composition, especially in the case of HS. Some of the proteins that we found to be strongly bonded to graphene nanoflakes (albumin, immunoglobulins, complement, and apolipoproteins) are also reported to have good affinity for other carbon-based nanomaterials such as carbon nanotubes^45–48^; however, the data at the present stage do not allow to conclude a general trend for carbon-based materials in a competitive environment such as full serum. Apolipoprotein A-I, the major component of high-density lipoprotein (HDL), was found to be highly abundant on the nanoflakes exfoliated with HS. It is also interesting to note that apolipoprotein B100 (the major component of low-density lipoproteins, LDL) was nearly absent even though it is commonly found in the corona of several nanoparticle types, such as silica and amine-modified polystyrene nanoparticles^49, 50^. However, we now understand that the composition of the corona may reflect little on the abundance of specific recognition motifs present in endogenous HDL, and therefore consider that question in more detail later in the paper.

HDL is a complex of small lipoproteins containing an outer-shell layer of phospholipids, free cholesterol stabilized by apolipoproteins (apoA-I for the 70%), and a hydrophobic lipid core of cholesterol esters and triglycerides^51, 52^, and plays a major role not only in the lipid metabolism (cholesterol efflux) but also in the innate immunity^53^. Therefore, this result is even more interesting, considering the reported affinity between graphene (and GO) surface and lipids. It has been reported that both the materials can promote the formation of supported lipid bilayers^54^, self-organization of phospholipids on their surface^55^, or vesicles in lipid layers^56^. Moreover, they can promote the disruptive extraction of phospholipid molecules from the lipid bilayers by phospholipid interaction onto its own surfaces^57^.

The presence of apolipoprotein A-I could suggest the binding of intact HDL lipoprotein complexes onto the nanomaterial, but whatever the source, depending on how the apolipoprotein A-I is presented, combined with the flat shape of graphene nanoflakes, suggests that they might be biologically recognized as HDL complexes, though without the capacity to modify its structure that HDL possesses^58^. Various studies in the past have suggested that HDL elasticity and the capacity to adopt such flattened structure could affect cellular uptake, as well as interendothelial transport^59^. Whether such capabilities could be transferred onto graphene flakes in the relevant media is unknown, but it will at least be of interest to understand the nature of the dispersion and the biological presentation.

### Graphene nanoflakes dispersion characterization

**Fig. 2:**
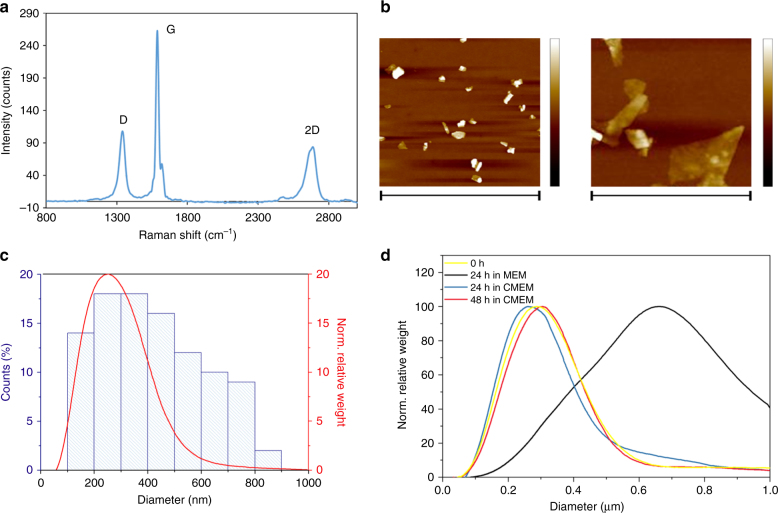
Complete characterization of exfoliated graphene nanoflakes in 100% v/v HS. **a** Raman spectrum, laser excitation at 514 nm, showing the typical D, G, and 2D peak at ~1350 cm^−1^, ~1580 cm^−1^, and ~2750 cm^−1^, respectively, is consistent with a graphene material; **b** AFM scans of 5 µm × 5 µm (left) and 1 µm × 1 µm (right) showing the nanoflakes topography. The presence of proteins on the nanoflakes can be noticed at higher magnification. High scales are, respectively, 50 nm (left) and 30 nm (right). **c** DCS analysis and AFM larger lateral size statistical analysis of graphene nanoflakes. The two methods resulted reasonably consistent between them, giving a main peak at about 200–300 nm. **d** Graphene nanoflakes stability over time (24 and 48 h) in cell culture media with (CMEM) and without (MEM) proteins as measured by DCS. The curves show that in the presence of protein-supplemented media, the graphene dispersion keeps its colloidal stability ove time with negligible aggregation

To characterize the obtained graphene nanoflakes dispersions, Raman spectroscopy, atomic force microscopy (AFM), differential centrifugal sedimentation (DCS), and ultraviolet/visible (UV/Vis) extinction spectroscopy were used, as shown in Fig. 2 and in Supplementary Fig. 5 and 6. Raman spectroscopy is one of the most common characterization methods for graphite and graphene^60–62^. Figure 2a depicts the Raman spectrum for graphene nanoflakes dispersion (dried droplet), showing the characteristic D, G, and 2D bands typical of graphene/graphite. Graphene nanoflakes edges activate the D band at ~1350 cm^−1^ (in the absence of other plane defects), while the number of graphene layers modify the shape and intensity of the 2D band (~2750 cm^−1^). Monolayer graphene presents a single narrow 2D peak with twice the intensity of the G band (~1580 cm^−1^)^60–62^. The overall spectral pattern (D/G intensity and shape and relative intensity of the 2D band) is consistent with the presence of few-layer graphene^41^. More spectra and their *I*_2D_/*I*_G_ ratios are shown in Supplementary Fig. 7.

AFM confirmed the presence of few-layer graphene nanoflakes (Fig. 2b; Supplementary Fig. 6). Lateral size distribution ranged between 100 and 800 nm, and a main population of nanoflakes around 200–300 nm was found after the analysis (Fig. 2c).

Although the size distribution given by DCS is not formally correct in the case of nonspherical particles, still it can produce very consistent and repeatable results even when applied to graphene^63^, allowing to distinguish different populations and to detect aggregation.

DCS and AFM showed comparable results for size distributions with a maximum peak around 250 nm for graphene exfoliated in 100% v/v HS after 2 h of ultrasonication (Fig. 2c). These size-distribution data were in agreement to what has been found for graphite exfoliation in surfactants^64, 65^. By further AFM analysis presented in Supplementary Fig. 6, thicknesses up to 25 nm are found. Taking into account the thickness of the protein layer, and the typically observed overestimated measured AFM thickness of liquid-exfoliated nanoflakes compared to the theoretical thickness^64, 66–68^, the number of graphene layers was estimated to range between 2 and 10 layers.

Despite the clear challenge for such a complex suspension, the ζ potential for the dispersions at pH = 7 was measured, resulting in −28 mV. In such complex mixtures, the meaning and validity of zeta potential should be considered with some caution. A more pertinent point is that the effects that stabilize the nanomaterials in the presence of proteins (either in serum, or isolated hard-corona complexes) are believed to be similar to those that stabilize the protein solution: a mixture of charge (not usually the primary enabling mechanism for dispersion) and hydrogen bonding, as well as entropic interactions (e.g., water ordering) and other forces are believed to play a significant role.

It is frequently observed that changing the media composition can impact the colloidal stability of the nanomaterial; therefore, it is essential to assess the nanomaterial stability in a given biological media over time to translate the material toward in vitro testing. The stability of graphene nanoflakes was tested in the biological medium serum-free minimum essential medium (MEM) and supplemented with 50% v/v of serum (complete MEM or CMEM).

DCS measurements were performed after 24 h of incubation at 37 °C in MEM and CMEM, and any shaking was performed during the incubation or prior to the analysis, to be closer to in vitro exposure conditions. In serum-free MEM after 24 h, a deposit of flakes can be clearly noticed, and almost nothing can be measured in the supernatant. After few seconds of vortexing, the sample can be measured, but it results in larger aggregates (Fig. 2d, black curve). In 50% v/v serum-supplemented medium (CMEM), the dispersion remained very stable over 24 h, without the need for vortexing, as can be seen in Fig. 2d (blue curve). After 48 h, the flakes can be seen to sediment on the bottom of an Eppendorf™ LoBind microcentrifuge tube, but after few seconds of vortexing, they can be measured at DCS, resulting in the same stable distribution (Fig. 2d, red curve). We can therefore conclude that the protein exfoliated graphene is stable up to 24 h in serum- supplemented medium, and this is the first time that the colloidal long-term stability is demonstrated for graphene biological dispersion.

### Protein functionality and availability of key epitopes

It is now widely accepted that the slowly exchanging biomolecular part of nanomaterial–biomolecular complexes determines the biological identity of the nanomaterial via the presentation of key recognition motifs in the corona that interact with relevant receptors^28^. The peripheral surface of the particles is therefore dependent on the conformation and orientation of biomolecules within the protein corona, which can be studied by methods currently under development^25, 27, 28^. Most of these methods use recognition (such as antibodies) and reporting functions (say gold nanoparticles (GNPs) or QDs) that recognize sites close to the reported recognition domains for relevant receptors, but in any case, they are capable of giving information on the general interface organization. Prior to the experiment, we made sure that the effect of prolonged sonication did not affect the protein conformation. To this aim, we exploited tryptophan fluorescence emission and circular dichroism as an indicator of the tertiary structure for some proteins. The results reported in Supplementary Fig. 8 showed a negligible effect of bath sonication on the proteins tertiary structure for the conditions used in this paper.

**Fig. 3:**
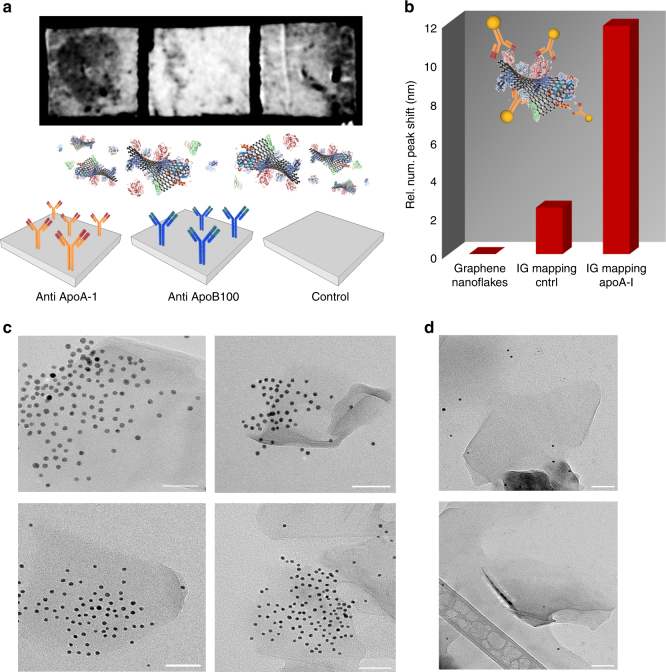
Immunometric mapping of the relevant epitope on apoA-I. **a** Immuno dot blot and scheme of the interaction for graphene exposed to a monoclonal antibody anti-apoA-I, monoclonal antibody anti-apoB100, and empty PVDF membrane. When exposed to a monoclonal antibody anti-apoA-I, a dark dot is visible on the PVDF membrane, indicating that the recognition has occurred. **b** In situ (without washing) differential centrifugal sedimentation (DCS) analysis of the graphene nanoflakes before and after incubation with IG anti-apoA-I and IG control. The graph represents the increase of diameter when the recognition occurs indicated as a shift of the DCS peak maximum normalized on the pristine graphene nanoflakes dispersion. **c** TEM micrographs showing the immunogold mapping^27^ technique applied to graphene nanoflakes exfoliated with HS. Monoclonal antibody anti-apoA-I was functionalized with 4-nm gold nanoparticles and incubated with graphene nanoflakes. When the recognition occurs, the gold nanoparticles can be seen on top of the flakes in the TEM micrographs, thanks to their higher electron density. **d** TEM micrographs of the control sample: human serum albumin was used in place of the monoclonal antibody anti-apoA-I to prove the specificity of the interaction. A negligible portion of gold nanoparticles is visible in these TEM micrographs. Scale bars are 50 nm

Preliminary evidence on the availability of exposed epitopes of interest on the graphene nanoflakes surface was obtained by immuno dot-blot assay, as described in Methods. The results are reported in Fig. 3a, and expectations from macroscopic proteomics (the predominance of apolipoprotein A-I in the exfoliated flakes and the absence of apolipoprotein B100) were confirmed using monoclonal antibodies anti-apoA-I and anti- apoB100.

Since the exfoliated graphene produces a black spot if adsorbed onto the polyvinylidene fluoride membranes (PVDF), making the fluorescence of the secondary antibody undetectable, a different strategy was used for this immunoassay. The dark color of graphene was therefore exploited as a signal, and a dark circle can be clearly seen when the interaction with antibodies occurs (Fig. 3a). An empty PVDF membrane was used as a control, and no adsorption of graphene flakes onto the membrane was found after incubation (Fig. 3a). A second control was represented by a functionalization with monoclonal antibody anti-apoB100, and the results confirmed the specificity of the interaction.

To understand the likelihood of biological recognition of exfoliated graphene nanoflakes (which after all occurs on a particle-by-particle basis), we aimed to map out the relevant recognition motifs at the surface of graphene by using recently reported immunomapping methods^25, 27^. In this paper, we used two different immunoprobes (called immunogold, IG or immuno-quantum dots, IQDs) that consisted of GNPs or QDs with a nominal diameter of about 4 nm functionalized with a specific monoclonal antibody; in this case, again the monoclonal anti-apoA-I was able to recognize amino acids 113–243 of apoA-I of human origin (proxy for the HDL receptor-binding domains)^69^.

It should be noted that the application of these approaches for graphene is more challenging than for many other nanomaterials to which it is routinely applied, given the peculiar size, thickness, and shape variety of the nanoflakes, but it can still be carried out in order to collect a series of evidences. Moreover, significant care must be taken in preparing these antibody constructs to avoid non-specific binding. The graphene nanoflakes exfoliated with HS were exposed to a large excess of IG and IQDs (detailed in the Methods section), and then washed an increasing number of times, following the procedure described elsewhere^25, 27^. Negative controls (Fig. 3d; Supplementary Fig. 9b) suggest that the only IG or IQDs remaining are those bound by biologically mediated recognition.

Thus, as shown in Fig. 3b, first, we do recognize a shift in the main peak in DCS after incubation with immune probes, suggesting in situ antibody recognition across the whole distribution of particulates.

In the case of IG mapping, as shown in Fig. 3c and Supplementary Fig. 9, direct imaging by transmission electron microscopy (TEM) showed the presence of 4-nm GNPs retained on the surface of the flakes after washing. As a negative control (IG_cntrl), the nanoflakes were also exposed to GNPs functionalized with HSA and very little non-specific adsorption was found (Fig. 3d; Supplementary Fig. 9b).

To confirm these data, IQDs were also used to detect the availability of apoA-I functional epitopes, as described in Supplementary Information. The emission spectrum (Supplementary Fig. 10) showed a fluorescence emission peak corresponding to the emission of the QDs retained after washing. The spectrum was normalized over the emission of the negative control (QDs functionalized with albumin), therefore, it is representative only of specific interactions.

All these evidences suggested the widespread surface presentation of the relevant apoA-I epitopes, able to promote specific recognition by, for instance, suitable cell receptors.

The estimation of an average number of exposed epitopes per particle is extremely challenging in the case of graphene flakes. The wide distribution of the flakes in terms of size and shape made it very difficult to reasonably estimate the number of particles. Also, the drying effect on the TEM grid made it very difficult to isolate a statistically significant number of single flakes. However, based on the samples mass concentration, and the QDs emission intensity, it was possible to estimate about 1 × 10^14^ functional apoA-I epitopes per mg of exfoliated graphene nanoflakes, as detailed in Supplementary Fig. 10.

## Discussion

The preparation of biologically relevant dispersions of nanoparticles has grown in significance and substance as the route to developing our understanding of the nanomaterial–biological interactions, prior to degradation of such layers, typically in lysosomes. Progressively, we have understood that, besides the size and shape, such particles act as scaffolds on which biomolecular recognition motifs (derived from the exposure medium) are presented, and that these factors likely combine to determine receptor interactions, and thereby likely various aspects of liver clearance and other organ-level interactions. Furthermore, the typical surface-free energies of nanoparticles are sufficiently large (and graphene lies at the larger end of this scale) that for many practical purposes of biological study, the corona is essentially fixed after its initial formation, so that the biomolecular components from which the corona is formed become a critical element of the study.

The evidence we have accumulated from such samples here shows that in sharp contrast to many other nanomaterials, graphene nanoflakes present a negligible proportion of apoB100-type recognition motifs, but are rich in effective apoA-I presentation. This certainly suggests that graphene could have distinct early biological interactions, both at the cell and organ level. One should be cautious though. Even if such differences are real, they will not be superficially obvious. That is, graphene will still be accumulated in cells, and within organs (reticuloendothelial (RES) system, including liver and spleen), as with all other nanomaterials, but the detailed mechanisms, cell types, and processes by which this occurs could be different. That is something which will have to be investigated in some detail.

In any case, a full understanding of the biological identity of graphene will likely include its shape, size, and the fact that certain components (in this case apoA-I derived from HDL) and potentially other less-abundant proteins, are presented at the surface. The simple, inexpensive, and scalable way for the graphene production and the resulting dispersions presented here are reproducible and very stable over time, and constitute the basis of a rational approach to the formation of biologically relevant samples for the study of graphene.

## Methods

### Preparation of the graphene dispersion and size selection

Dispersions of graphene in aqueous solution were prepared by 1–4 h of ultrasonication of 10% w/v of natural flake graphite (Asbury, grade 3763) dispersed in a solution of serum at different concentrations. The ultrasound-assisted exfoliation was performed using both FBS and HS at the concentrations of 10%, 50%, and 100% v/v in PBS. FBS was purchased from Gibco by Life Technologies (catalog number 10270), and HS off the clot was purchased from Millipore (catalog number S1-100).

For the exfoliation, a bath sonicator Fisherbrand FB11207 was used at the frequency of 37 kHz and 100% of power. The temperature was kept around 15 °C by a mixture of water and ice (70:30) in the bath which was frequently replaced.

The centrifugation steps for size selection were performed using an Eppendorf 5810 R centrifuge. An Eppendorf 5410 R centrifuge with a fixed rotor 1195-A and a 1.5-mL Eppendorf™ LoBind microcentrifuge tube were used for the washing procedure. Size selection and washing procedures are described in detail in Supplementary Methods.

To measure the mass concentration of exfoliated graphene nanoflakes, an ultrabalance Sartorius, Cubis® and aluminum boat (Lüdi Swiss) were used.

### Characterization

The final dispersions have been fully characterized by DCS, Raman spectroscopy, AFM, and absorption spectroscopy, as shown in Fig. 2 and in Supplementary Fig. 5 and 6.

DSC experiments were performed with a CPS Disc Centrifuge DC24000 (CPS Instruments). A total of 100 μL of sample were injected in an 8–24% PBS-based sucrose gradient. Density values of 1.75 g × mL^−1^, refractive index of 2.377, and a non-sphericity factor of 3 were used. The rotational speed of the disk was set to 18,000–20,000 rpm.

AFM was carried out in tapping mode on a Bruker Innova system using MPP-11123-10 tapping-mode probes. A drop of the dispersion (10 μL) was deposited on a pre-heated (120 °C) Si wafer with a 300-nm-thick SiO_2_ layer. This technique accelerated the drying, minimizing the aggregations. The wafer was rinsed with Milli-Q water after the deposition.

UV–Vis extinction spectra were recorded using a Varian Cary 6000 UV-Vis spectrophotometer in a 1-cm path quartz cuvette.

Raman spectroscopy was performed using a Horiba Jobin Yvon LabRAM HR800 with a 514-nm excitation laser in air under ambient conditions. The Raman emission was collected by ×100 objective lens (N.A. = 0.8). To avoid sample heating, Raman experiments were carried out at 10% of maximum laser power (<2 mW). The spectra were recorded on a dried droplet and 50 spectra were averaged to obtain a representative mean.

For electron microscopy analysis, a drop of concentrated sample was deposited onto a glow-discharged holey film grid (TED PELLA INC. Ultrathin Carbon Film on Lacey Carbon Support Film, 400 mesh, Copper). The grid was kept in a humidified environment for 45 min and then rinsed with three drops of Milli-Q water. The grid was subsequently dried and visualized using FEI Tecnai G2 20 Twin TEM.

ζ potential of the NPs suspension was measured by Zetasizer Nano ZS by using a disposable capillary zeta cell. ζ potential measurements reported are an average of five independent measurements, with each measurement consisting of an accumulation of ten runs.

Circular dichroism spectra were recorded on a J-810 (JASCO) Spectropolarimeter. The spectra were recorded at 25 ± 0.2 °C using a quartz cuvette of 1-mm path length (Hellma Analytics); the temperature was maintained by a Peltier thermostat, and the spectra were averaged over eight measurements.

### 1D SDS-PAGE

After the washing procedure (described in Supplementary Methods), the proteins surrounding the graphene were denatured by boiling for 5 min in blue loading buffer composed of 62.5 mM Tris-HCl (pH 6.8 @ 25uC), 2% (w/v) SDS, 10% glycerol, 0.01% (w/v) bromophenol blue, and 40 mM DTT. After this procedure, the corona proteins were denatured and coated with SDS surfactant (which provides them with negative net charge). The samples were loaded in a 10% polyacrylamide gel (1D SDS-PAGE), and separated by size upon application of an electric field using a Mini-PROTEAN Tetra electrophoresis system from Bio-Rad. A constant voltage of 130 V was applied for about 45 min of the electrophoretic run. In order to visualize the protein bands, all the gels were stained using 2D-SILVER STAIN-II reagents (Cosmobio Co.,Ltd) prior to scanning under white light using a G:Box Chemi XT4 (Syngene). Uncropped scans of the gels are reported in SI.

### Mass spectrometry

The proteins on the samples have been denatured and separated from the graphene flakes in a 1D SDS-PAGE, and allowed to run for about 10 min until the buffer line was about 1 cm past the interface between the stacking gel and the separation gel. This was carried out to condense all the proteins into a single sample for MS analysis, thereby avoiding gel fractionation. The SDS-PAGE gel was stained using Coomassie stain in order to visualize the proteins. The big gel bands in each lane stained with Coomassie were excised using a sterile scalpel and transferred to a clean 0.5-mL sample tube which had been pre-rinsed with acetonitrile, and the proteins were digested in the gel by trypsin digestion. At the end of this process, the samples were resuspended in 0.1% w/w formic acid prior to MS analysis by electrospray liquid chromatography.

All samples were run on a Thermo Scientific LTQ ORBITRAP XL mass spectrometer connected to an Exigent NANO LC.1DPLUS chromatography system incorporating an auto-sampler.

The raw mass spectral data have been searched against bovine and human protein database and analyzed using two different software packages (Peaks 7.5 and MaxQuant 1.4.1.2)^70^ in order to obtain a semiquantitative estimation of the relative protein coverage (%) for each protein in the samples. The method of spectral counting (SpC) representing the total number of the MS/MS spectra for all the peptides attributed to a matched protein was mainly used. By applying Eqn (1), the spectral counts related to each protein's identity were then normalized (NSpC) to the relative protein mass and expressed as the relative protein coverage (%)

1 $${\mathrm{NSpC}}_{\mathrm{k}} = \frac{{({\mathrm{SpC}}/M_{\mathrm{w}}){\mathrm{k}}}}{{\mathop {\sum}\nolimits_{i = 1}^n {({\mathrm{SpC}}/M_{\mathrm{w}}){i}} }} \times 100$$

where NSpC_k_ is the percentage NSpC for protein k, SpC is the identified spectral count, and *M*_w_ is the molecular weight in KDa for protein k. This correction takes into account the protein size in order to evaluate the real contribution of each protein.

The obtained results have been compared with the method of the label-free quantification^71^ performed by MaxQuant. This method, able to accurately and robustly quantify small fold changes on a proteome scale, has the prerequisite that a majority population of proteins exists that is not changing between the samples, therefore, it has been applied to the samples incubated with the same serum at different percentages.

### Immuno dot blot and immunometric mapping

For the immuno dot blot and immunolabeling experiments, apo A-I antibody (A 5.4), cat. num. sc-13549, mouse monoclonal IgG, and apoB100 antibody (A-6), cat. num. sc-393636 from Santa Cruz Biotechnology were used.

For the immuno dot blot, the monoclonal anti-apoA-I and anti-apoB100 antibodies were spotted on the PVDF membrane at the concentration of 2 μg mL^−1^. A membrane without an antibody was used as a further negative control. The blots were blocked in 5% skimmed milk in PBS for 1 h at room temperature, washed three times in PBS, and incubated with the graphene nanoflakes at the concentration of about 20 μg mL^−1^. After 1 h of incubation with the exfoliated graphene solution in PBS at room temperature, the blots were washed five times for 10 min, dried, and scanned under white light using a G:Box Chemi XT4 (Syngene) to detect the presence of graphene bounded to the primary antibody in the spot on the membranes.

For IG and IQDs preparation, 4-nm GNPs and 4-nm CdTe QDs were synthesized as described elsewhere^26^. For antibody (IgG) conjugation, a carbodiimide-based strategy was adopted for both GNPs and QDs, with small differences. Briefly, 0.2 nmol (1.65 nmol) of GNPs (QDs) were mixed with 0.8 mg of EDC and 1.6 mg of Sulfo-NHS in MES buffer, pH 6.5 (PBS pH 7.4), and the mixture was incubated at 37 °C for 30 min. The activated GNP (QD) solution was purified from the unreacted EDC/Sulfo-NHS by passing it through a PD-10 column (GE Healthcare Life Science, Ireland) using MES (PBS) as exchange buffer. Then IgG was added to the GNPs (QDs) in a 0.8:1 molar ratio and the mixture was stirred at 37 °C for 1 h. The ratio Ab/GNP (Ab/QD) was optimized to get one antibody per nanoparticle. Subsequently, the excess of activated carboxylic groups was blocked by the addition of 4-aminophenyl-β-D-galactopyranoside (5 mg·mL^−1^), and the mixture was incubated overnight. IG (final concentration 90 nM) and IQDs (final concentration 500 nM) were stored at 4 °C.

For IG mapping experiments, 20 µL of graphene nanoflakes dispersion (conc. 200 µg  mL^−1^) were incubated with 100 µL of IG at 37 °C for 1 h and then washed two times with fresh PBS by centrifugation at 20,000 × *g* for 10 min. Then 20 µL of BS(PEG)9 (bis-N-succinimidyl-(nonaethylene glycol) ester, 21582 (ThermoFisher) were added to the sample and incubated at 37 °C for 2 h, and then washed three times with fresh PBS by centrifugation at 10000 × *g* for 10 min prior to the preparation of the TEM grid.

For IQDs mapping experiments, 10 µL of graphene nanoflakes dispersion (conc. 700 µg mL^−1^) were incubated with 80 µL of IQDs for 1 h at 37 °C under shaking and then washed two times with fresh PBS by centrifugation at 8000 × *g* for 10 min. The concentration of graphene flakes after the washing steps was measured by UV–Vis spectroscopy. Fluorescence spectroscopy measurements were performed with a Horiba Jobin Yvon Fluorolog-3 fluorimeter using a 45-μL quartz ultra-micro cuvette of 3-mm path length (Hellma Analytics). For each sample, emission spectra were recorded using *λ*_ex_ = 375 nm as the excitation wavelength. QDs functionalized with bovine serum albumin (QD-BSA) were used as control for unspecific binding.

### Data availability

Data supporting the findings of this study are available within the article (and its Supplementary Information files) and from the corresponding author upon reasonable request.

## Electronic supplementary material

Supplementary Information

Description of Additional Supplementary Files

Supplementary Data 1

Supplementary Data 2
