# Supplementary material for: Biological recognition of graphene nanoflakes
Source: Nat Commun. 2018 Apr 20;9:1577. doi: 10.1038/s41467-018-04009-x (PMC5910434; doi:10.1038/s41467-018-04009-x)
Supplement: Supplementary file 1 — Supplementary Information [file 41467_2018_4009_MOESM1_ESM.pdf]

Supplementary Information

Biological recognition of graphene nanoflakes

Castagnola et *al*.

Supplementary Figures

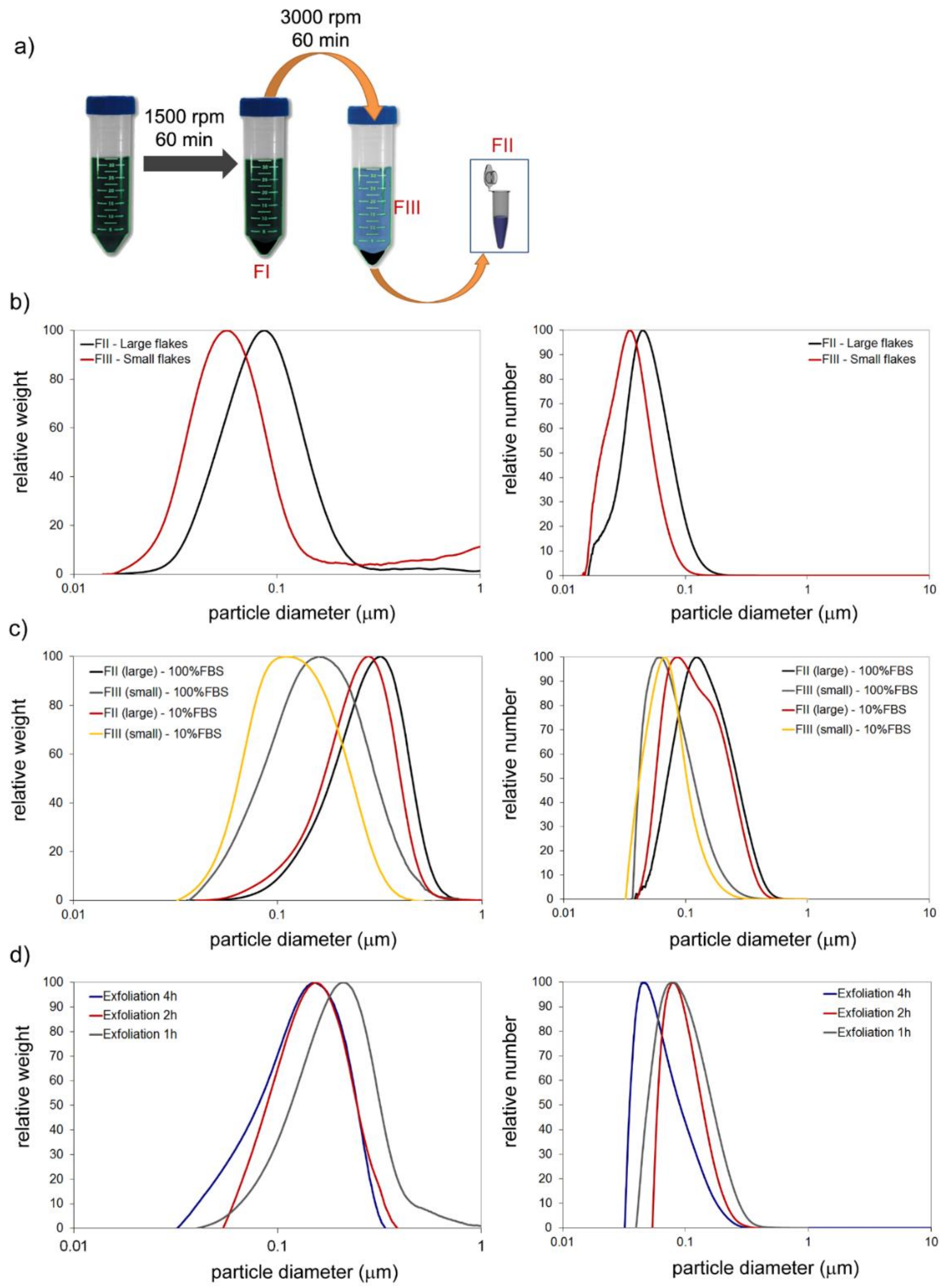

Supplementary Figure 1. **Graphene nanoflakes size selection procedure.** a) Scheme of controlled centrifugation steps used for the size selection. Laboratory plastics schemes adapted from Smart Servier Medical Art (https://smart.servier.com/). Examples of DCS analysis expressed in relative weight and number for b) fraction FII and FIII for graphene exfoliated with 10% v/v FBS and c) FII and FIII for graphene exfoliated with 10% and 100% v/v of FBS by 4h of ultrasonication. d) DCS analysis for graphene nanoflakes (FII) exfoliated with 100% v/v FBS by 1h, 2h and 4h of ultrasonication.

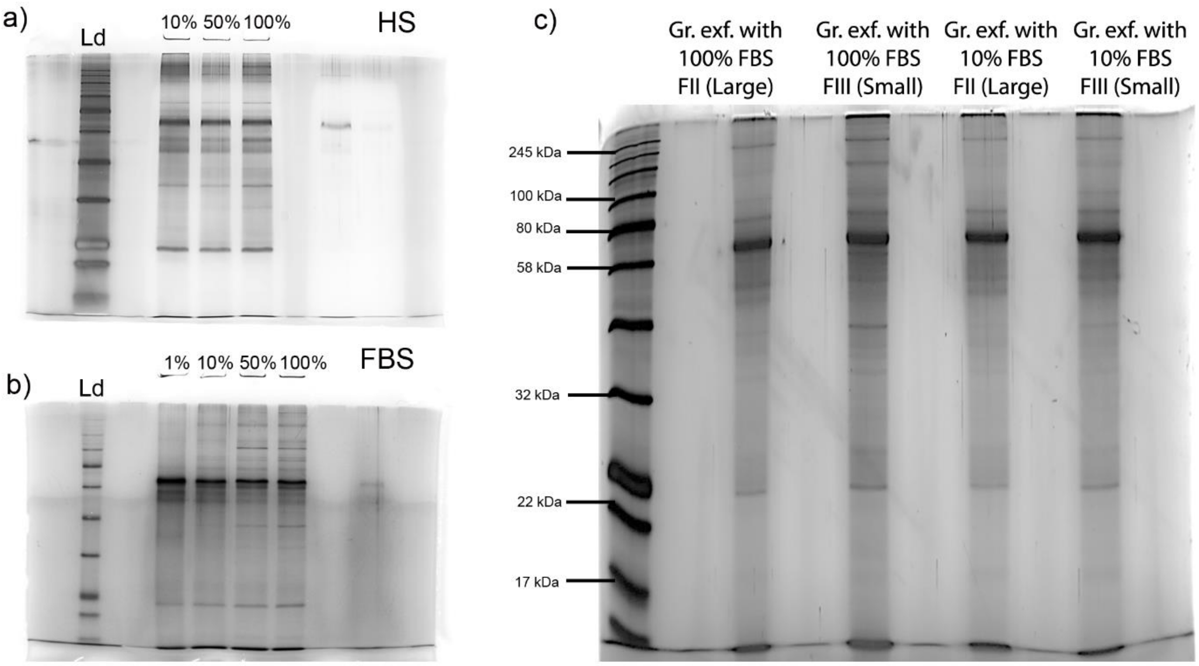

Supplementary Figure 2. Supplementary 1D SDS-PAGE. Uncropped 1D SDS-PAGE for graphene exfoliated in a) HS and b) FBS at different concentrations. b) 1D SDS-PAGE of graphene dispersions (FII, large nanoflakes and FIII small nanoflakes) exfoliated with both 10% and 100% of serum. The protein profiles appeared very similar for the two size ranges.

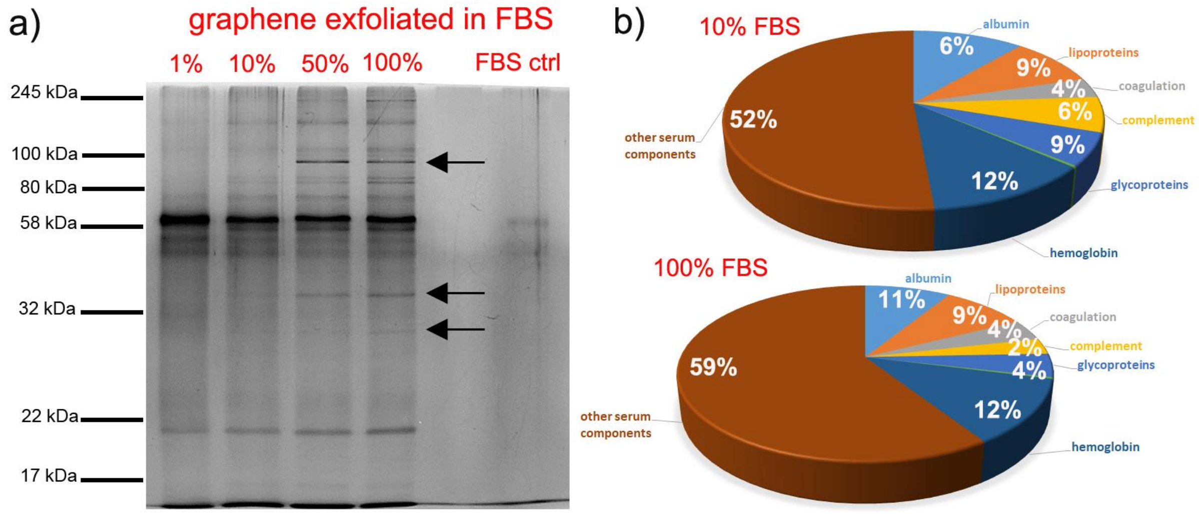

Supplementary Figure 3. **Proteomics analysis for graphene exfoliated with full FBS.** a) 1D SDS-PAGE for graphene exfoliated with 1%, 10%, 50%, 100% v/v of FBS. The black arrows highlight some differences in the protein profile as the starting concentration of FBS increases. In particular, a few differences emerged for molecular weight close to 80-100 kDa and 20-40 kDa (while no significant differences were observed for the different serum concentrations for the nanoflakes exfoliated in HS). b) Pie chart indicating the relative coverage of protein as identified by Mass Spectrometry onto the graphene flakes (exfoliated with 10% v/v and 100% v/v of FBS) and organised per protein function.

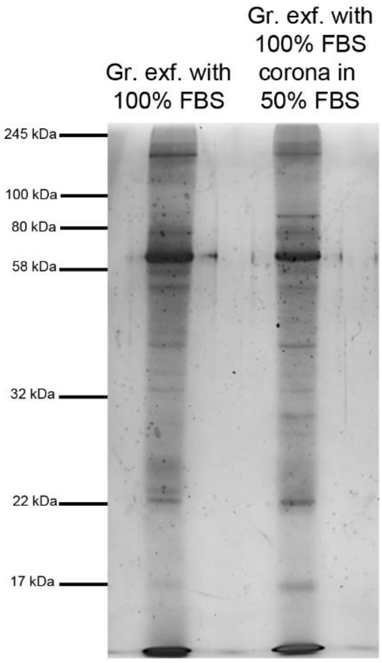

Supplementary Figure 4. Verification of protein displacement. 1D SDS-PAGE of graphene exfoliated with 100% v/v of FBS and the very same material subjected to further incubation with 50% v/v of FBS and washing. This is done in order to verify that the protein identified as hard corona are not displaced by other proteins present in the serum when the culture medium is supplemented to perform *in vitro* tests. Some differences in the bands intensity appeared in the zone of 90 kDa and 30 kDa but, the two profiles show the same bands around 60 kDa (albumin) and 45 kDa (apolipoprotein A-I).

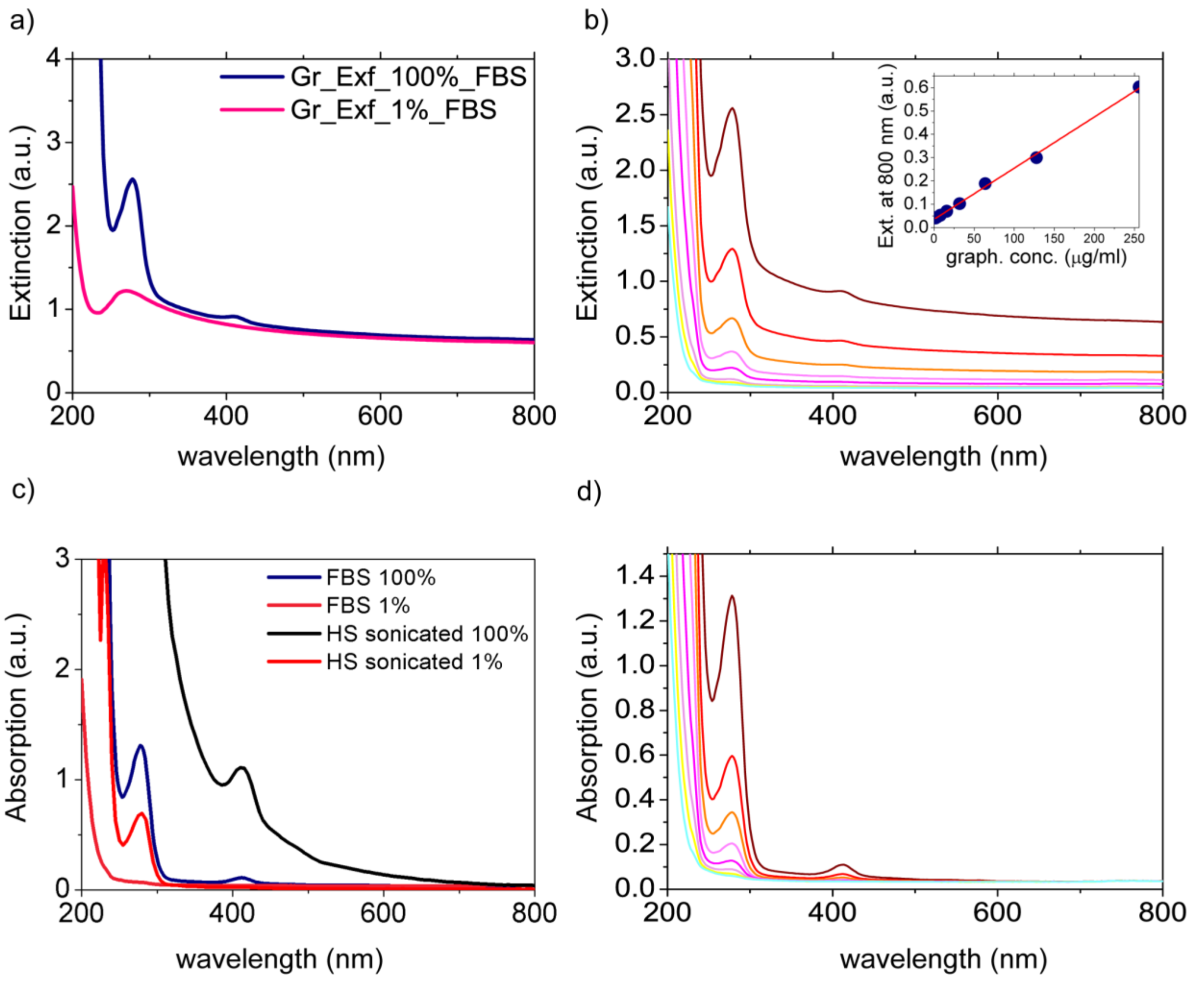

Supplementary Figure 5. **Photophysical characterization**. UV-Vis extinction spectra of the graphene solutions a) exfoliated with different serum concentrations. For a low protein content (1% v/v FBS) the π- π* peak around 264 nm is visible. Then, a plateau from 500 nm to 800 nm, due to the extinction of the graphene nanoflakes is visible. For a higher protein concentration (100% v/v FBS), the absorption peak related to the proteins start to appear around 278 nm and below 230 nm, covering the π- π* peak of graphene. b) Extinction for graphene exfoliated with 100% v/v of serum at different concentrations (wine to light blue curves indicates decreasing concentration, with sub-sequential 1:2 dilutions). The dependence of the extinction plateau between 500 and 800 nm from the concentration is not affected by the presence of the proteins. c) The absorption spectra of complete FBS at different % v/v (1% and 100%) and HS at different % v/v (1% and 100%) subjected to 2h bath ultrasonication. The ultrasonication did not induce protein aggregates that could generate appreciable scattering at 800nm. d) FBS at decreasing concentrations (with sub-sequential 1:2 dilutions) are shown for comparison purposes. A strong absorption of the proteins below 250

nm and the peak at 278 nm can be seen starting from a concentration of 10% v/v FBS and any absorption is found over 500 nm. Note that the contribution of scattering has been neglected.

For the proteomic study, the concentrations for samples of the same size distribution were normalised based on the optical extinction spectra. It was previously found that the extinction coefficient in the plateau region of the spectrum (800 nm) is widely size independent.[1] Therefore it is a suitable value for the normalisation of the graphene dispersions concentration.

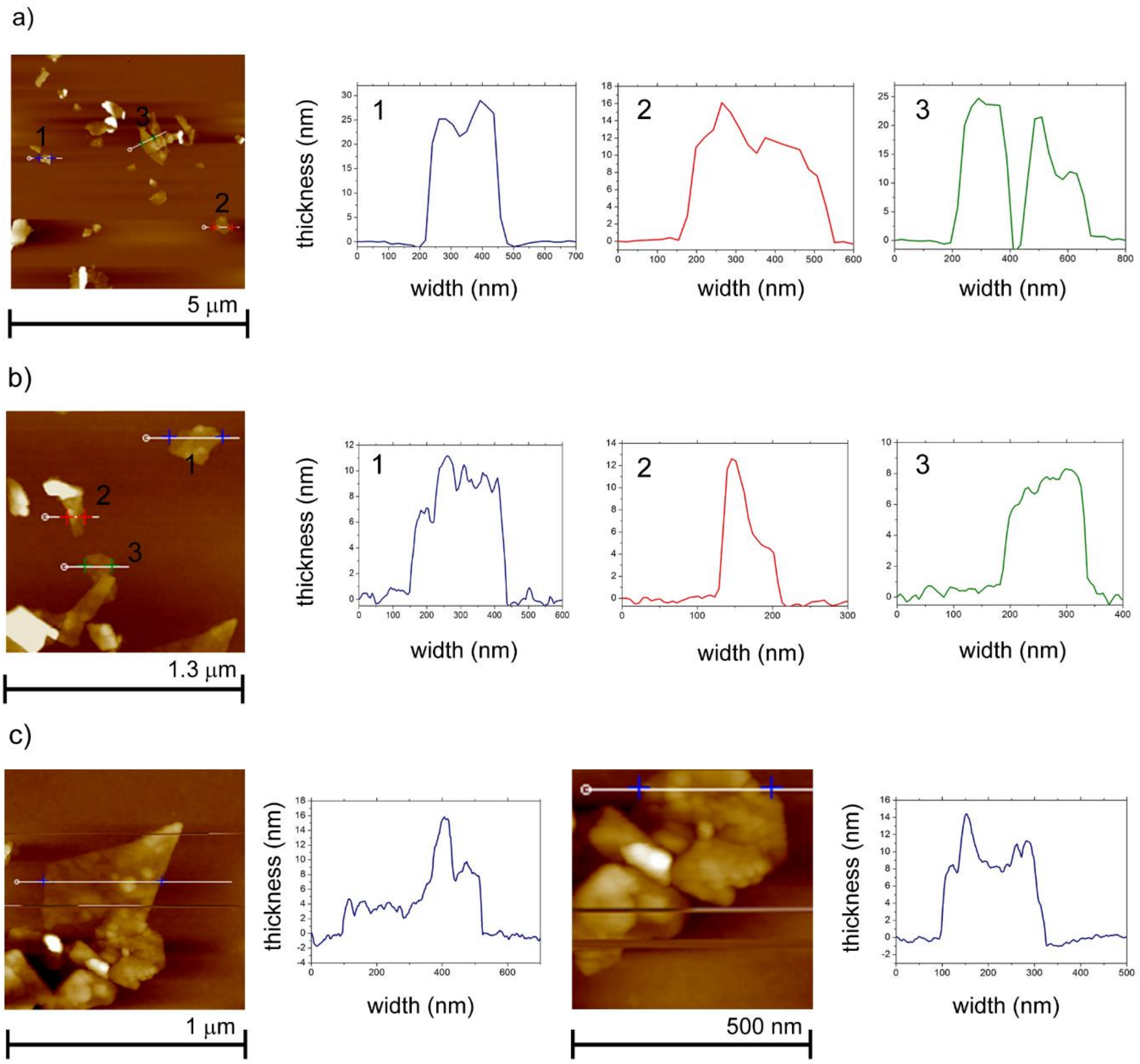

Supplementary Figure 6. **AFM analysis for the estimation of nanoflakes thicknesses**.

a) AFM section analysis showing two populations of thicknesses, one around 20 nm and another around 10 nm. c) Details of a flake on top of which some proteins peak can be distinguished, with a thicknesses of about 2-10 nm. Measured thicknesses showed a distribution of 8-25 nm. On top of the nanoflakes some structures are clearly visible, the thickness of which is compatible with the mean size of the serum proteins (2-10 nm). Therefore, taking into account the thickness of the protein layer, and considering the atomic thickness of graphene to be about 0.345 nm, the number of graphene layers would range between 6 and 50. Nevertheless, according to previous works, apparent measured AFM thickness from liquid exfoliated 2D materials seems to be always overestimated.[1-5] therefore the number of layers calculated dividing by the theoretical thickness of one graphene layer, might be overestimated. Considering that the height of terraces on incompletely exfoliated

nanoflakes was found to be around 1 nm for graphene exfoliated in surfactant,[1] we could estimate that the number of layers vary between 2 and 10 nm in this case. This result is more in agreement with the appearance of the Raman Spectra and more consistent with previously reported works.

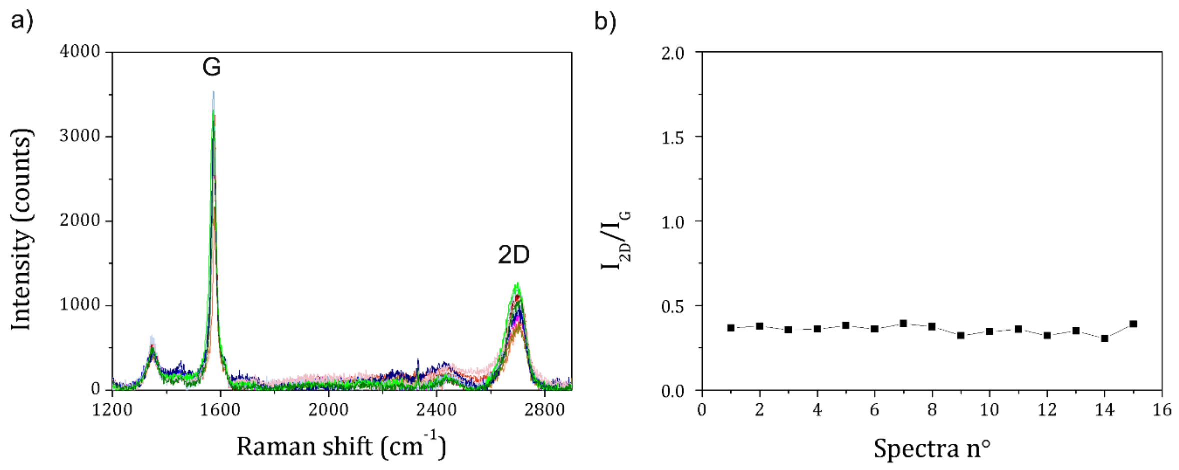

Supplementary Figure 7. **Raman spectroscopy characterization**. a) 15 representative Raman spectra recorded for graphene nanoflakes exfoliated with 50% v/v HS and b) relative ratio between 2D and G bands intensities. The ratio I_{2D}/I_G of these bands for high quality (defect free) single layer graphene is considered equal to 2.[6,7]

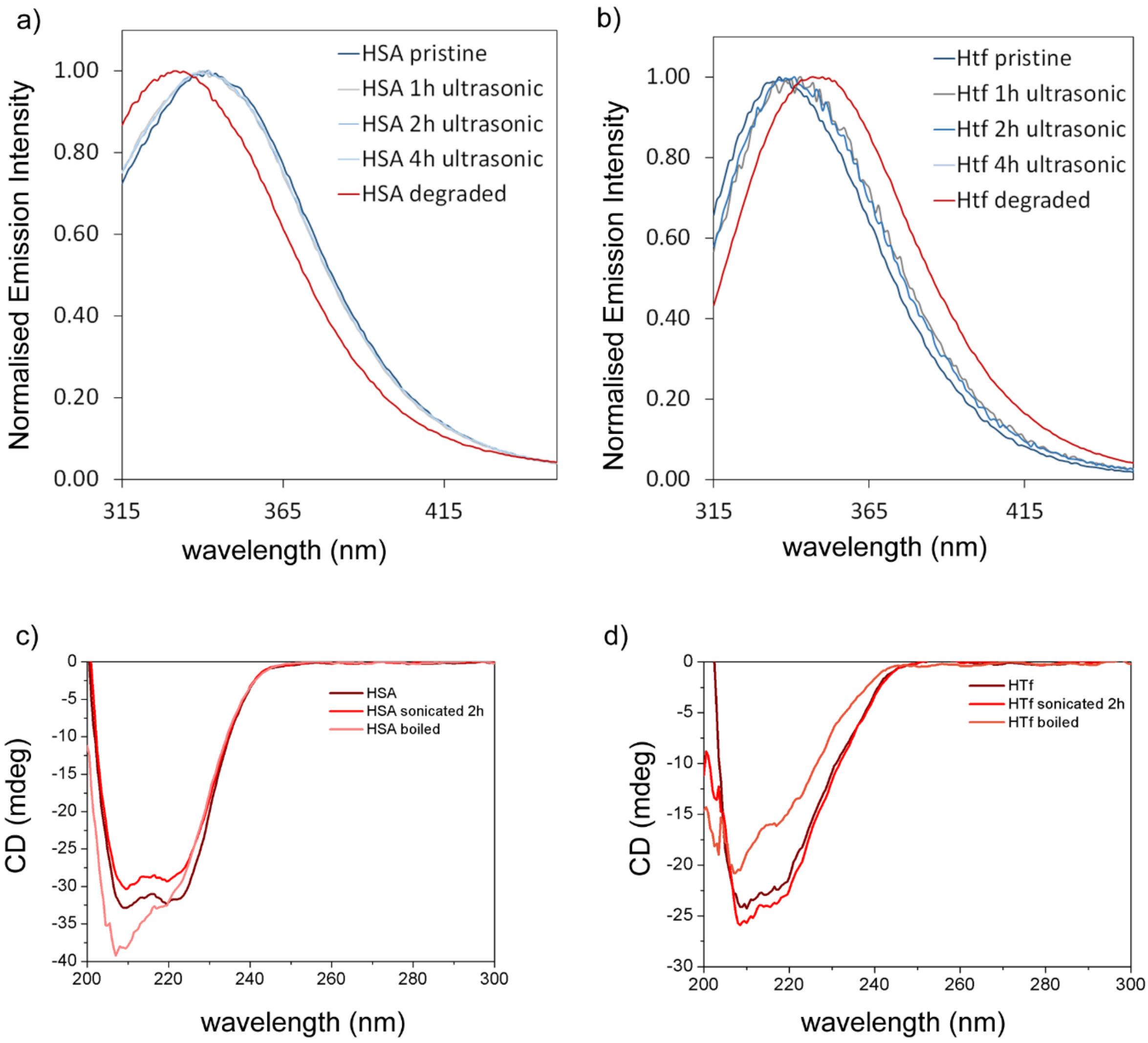

Supplementary Figure 8. Evaluation of the protein stability during the exfoliation procedure: a) and b) tryptophan fluorescence emission,[8] c) and d) circular dichroism. It has been reported that tip sonication can lead to protein denaturation.[9] The effect of ultrasonic bath has been reported to be less aggressive, since a major part of the energy is lost during the transmission of ultrasound through water and the glass of the sample flask.[10] Nevertheless, prior the "biosurface mapping" experiments, it was important to verify the effect of the sonication conditions on the conformational state of the proteins. From the literature, tryptophan fluorescence has been used previously as an indicator of tertiary structure for transferrin, since it contains 8 tryptophan residues at amino acids 27, 147, 283, 363, 377, 460,479 and 569.[8] The effect of 2h of ultrasonication on the same representative proteins was also assessed by circular dichroism.[11,12] Therefore two model proteins of different sizes were chosen: human holo-transferrin (HTf) and human serum albumin (HSA). The last one is the most abundant protein in serum and was also found highly abundant in the graphene corona.

a) Fluorescence emission of human serum albumin (HSA - blue curve), HSA after 10 min at 100°C to ensure the degradation (red curve) and after 1h (grey curve), 2h (turquoise curve) and 4h (light blue curve) in ultrasonic bath. b) Fluorescence emission of holo-transferrin human (HTf - orange curve), HTf after 10 min at 100°C to ensure the degradation (red curve) and after 1h (wine curve), 2h (turquoise curve) and 4h (light blue curve) in ultrasonic bath. c) Ellipticity of human serum albumin (HSA - wine curve), HSA after 10 min at 100°C to ensure the degradation (pink curve) and after 2h (red curve) in ultrasonic bath. d) Ellipticity of holo-transferrin human (HTf - wine curve), HTf after 10 min at 100°C to ensure the degradation (pink curve) and after 2h (red curve) in ultrasonic bath. From these exemplary test, the effect of the bath sonication on the proteins tertiary structure for the conditions used in this paper did not appear to affect the protein conformation.

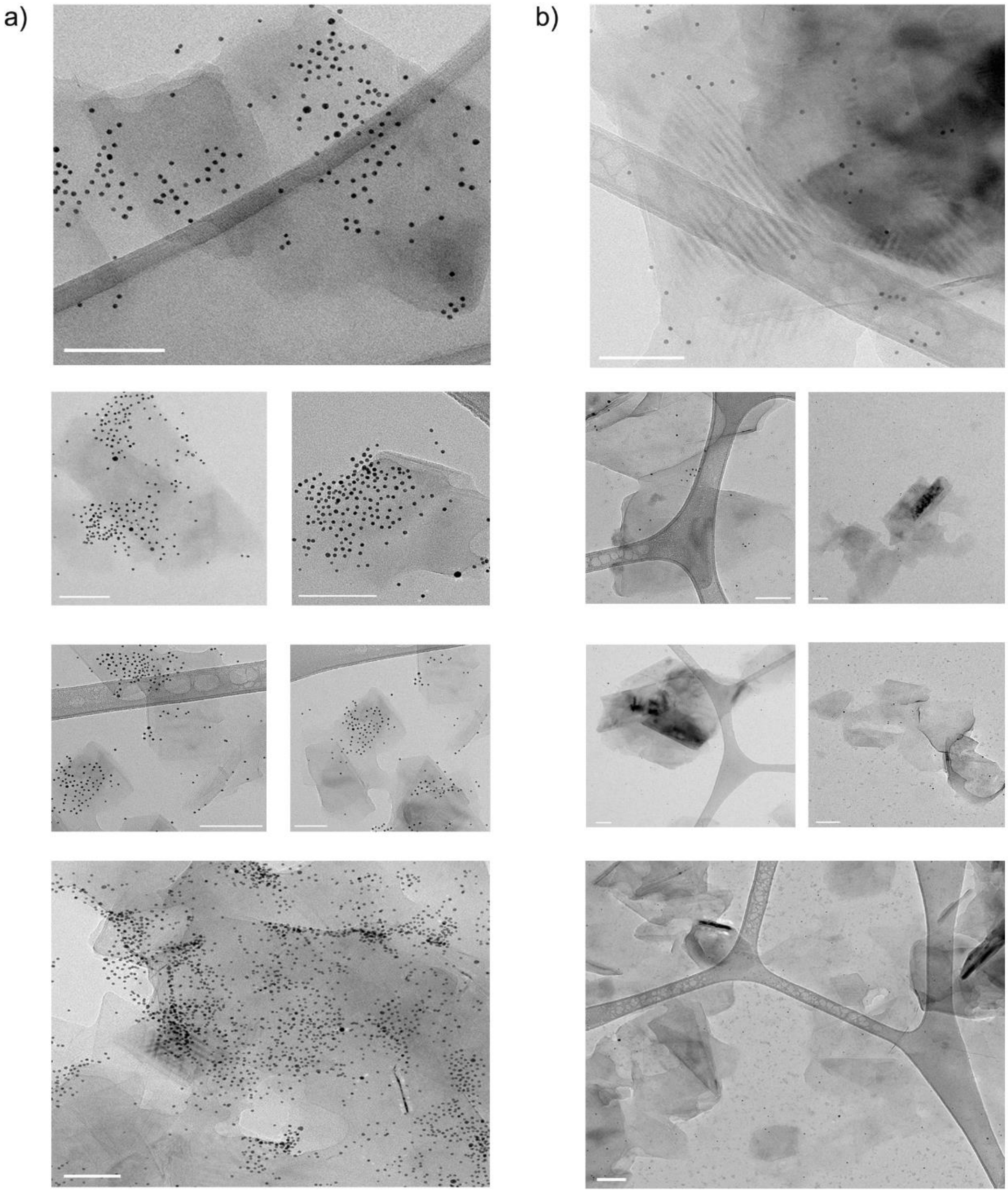

Supplementary Figure 9. Immunogold mapping of graphene nanoflakes. a) TEM micrographs showing 4 nm gold nanoparticles on top of graphene nanoflakes when the recognition occurs between apoA-I functional motifs and antibody anti apoA-I conjugated with the gold nanoparticles. b) TEM micrographs of the control sample: human serum albumin was used in place of the monoclonal antibody anti apoA-I to prove the specificity of the interaction. Scale bars are 100 nm.

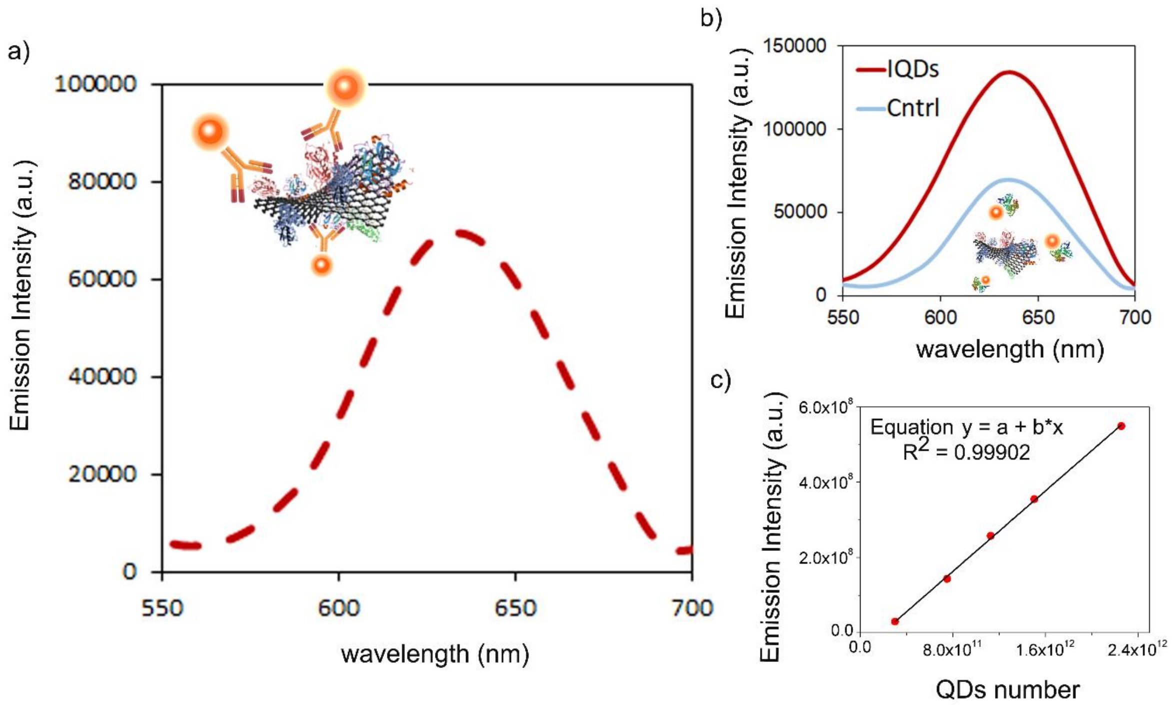

Supplementary Figure 10. ImmunoQDs mapping of graphene nanoflakes. In order to estimate the amount of apoA-1 epitopes on graphene nanoflakes, the photoluminescence peak of the IQDs treated samples was compared with a QD calibration curve, under the assumption that the total fluorescence of the sample with a certain number of IQDs is equivalent to the amount of fluorescence intensity of a solution of known QDs concentration. a) Emission intensity (normalised against the negative control) recorded after incubation of QDs functionalised with monoclonal antibody anti apoA-I (IQDs) with graphene nanoflakes. Washing steps were performed previous measurement to remove excess of unbounded IQDs. b) Emission intensity for IQDs after incubation with graphene nanoflakes and washing steps (red curve) compared to the emission intensity of the negative control (light blue curve) represented by QDs functionalised with albumin (unspecific interaction). c) Emission intensity for a growing number of QDs (calibration curve for the calculation of exposed epitopes per mg of graphene nanoflakes). Based on this data it was possible to estimate about $1{\cdot}10^{14}$ functional apoA-I epitopes per mg of exfoliated graphene nanoflakes.

Supplementary Tables

Supplementary Table 1. List of highly abundant proteins on the surface of graphene exfoliated in 100% Foetal Bovine Serum as identified by MS. The proteins are listed by relative abundance calculated by the method of normalised spectral counts (NSpC) as analysed by MaxQuant 1.4.1.2. In relation to the SDS-PAGE for the flakes exfoliated with FBS, some differences of the NSpC% could be found in particular for coagulation factor V (248 kDa), trombospondin 4 (105 kDa), apolipoprotein E (35 kDa) and transgelin-2 (22 kDa). The values for NSpC% almost doubled when graphene is exfoliated with 100%v/v of serum compared to 10% v/v.

ID	Protein name	Mol. Weight	NSpC %
P02768	Serum albumin	69.366	10.02
P02647	Apolipoprotein A-I	30.777	8.55
P02649	Apolipoprotein E	36.154	4.39
P04004	Vitronectin	54.305	4.30
P01009	Alpha-1-antitrypsin	46.736	3.40
P06727	Apolipoprotein A-IV	45.398	3.17
P68871	Hemoglobin subunit beta	15.998	3.10
V9GYE3	Apolipoprotein A-II	5.8767	2.53
P01024	Complement C3	187.15	2.41
P01008	Antithrombin-III	52.602	2.08
P04196	Histidine-rich glycoprotein	59.578	1.92
P10909	Clusterin	48.803	1.63
P02656	Apolipoprotein C-III	10.852	1.37
P0CG06	Ig lambda	11.237	1.32
P69905	Hemoglobin subunit alpha	15.257	1.30

Supplementary Methods

Graphene nanoflakes dispersion size selection

An initial centrifugation at 400 g for 60 min was performed to remove the un-exfoliated graphite and bigger aggregates (fraction FI). The supernatant was subjected to further centrifugation at 1700 g for 60 min. Again, the supernatant containing smaller nanoflakes (fraction FIII) and the sediment containing the larger nanoflakes were collected in fresh PBS (fraction FII). The two fractions were then washed by a series of 3 centrifugations at 20000 g for 15 min, where the supernatant is replaced each time by fresh PBS.

Graphene mass concentration calculation

To measure the mass concentration of exfoliated graphene nanoflakes an ultrabalance Sartorius, Cubis® and aluminum boat (Lüdi Swiss) were used.

The final samples were washed three times by centrifugation at 20000 g for 15 min and the PBS supernatant replaced with fresh MilliQ water prior ultrablance measurements. The aluminium boat was weighted 3 times to ensure weight measurement accuracy and statistical relevance then 50 μL of the concentrated colloidal dispersion was loaded into the boat. The sample was dried in a desiccator containing P_2O_5 for 24 h, the weight measured again 3 times, and the mass of the sample calculated. The number obtained × 20 represented the mass concentration of 1 mL of the sample.

Supplementary References

1 Backes, C. *et al.* Spectroscopic metrics allow in situ measurement of mean size and thickness of liquid-exfoliated few-layer graphene nanosheets. *Nanoscale* **8**, 4311-4323 (2016).

2 Backes, C. *et al.* Edge and confinement effects allow in situ measurement of size and thickness of liquid-exfoliated nanosheets. *Nature communications* **5** (2014).

3 Hanlon, D. *et al.* Liquid exfoliation of solvent-stabilized few-layer black phosphorus for applications beyond electronics. *Nature communications* **6** (2015).

4 Gibaja, C. *et al.* Few-Layer Antimonene by Liquid-Phase Exfoliation. *Angewandte Chemie International Edition* (2016).

5 Fan, X. *et al.* Controlled Exfoliation of MoS2 Crystals into Trilayer Nanosheets. *Journal of the American Chemical Society* **138**, 5143-5149 (2016).

6 Das, A., Chakraborty, B. & Sood, A. Raman spectroscopy of graphene on different substrates and influence of defects. *Bulletin of Materials Science* **31**, 579-584 (2008).

7 Hao, Y. *et al.* Probing Layer Number and Stacking Order of Few-Layer Graphene by Raman Spectroscopy. *small* **6**, 195-200 (2010).

8 Sharma, V. K. & Kalonia, D. S. Steady-state tryptophan fluorescence spectroscopy study to probe tertiary structure of proteins in solid powders. *Journal of pharmaceutical sciences* **92**, 890-899 (2003).

9 Joseph, D., Tyagi, N., Ghimire, A. & Geckeler, K. E. A direct route towards preparing pH-sensitive graphene nanosheets with anti-cancer activity. *RSC Advances* **4**, 4085-4093 (2014).

10 Mermillod-Blondin, F., Fauvet, G., Chalamet, A. & Creuzé des Châtelliers, M. A comparison of two ultrasonic methods for detaching biofilms from natural substrata. *International review of hydrobiology* **86**, 349-360 (2001).

11 Li, X. & Wang, S. Study on the interaction of (+)-catechin with human serum albumin using isothermal titration calorimetry and spectroscopic techniques. *New Journal of Chemistry* **39**, 386-395 (2015).

12 Sarzehi, S. & Chamani, J. Investigation on the interaction between tamoxifen and human holo-transferrin: determination of the binding mechanism by fluorescence quenching, resonance light scattering and circular dichroism methods. *International journal of biological macromolecules* **47**, 558-569 (2010).
