# Supplementary material for: Biological recognition of graphene nanoflakes
Source: Nat Commun. 2018 Apr 20;9:1577. doi: 10.1038/s41467-018-04009-x (PMC5910434; doi:10.1038/s41467-018-04009-x)
Supplement: Supplementary file 2 — Description of Additional Supplementary Files [file 41467_2018_4009_MOESM2_ESM.pdf]

Description of Additional Supplementary Files

File Name: Supplementary Data 1
Description: Complete list of proteins for graphene exfoliated with 100% FBS as identified by MS and analysed by MaxQuant by the method of normalised spectral counts (NSpC). Form this lists, keratin contaminations has been removed as well as proteins with NSpC < 0.1.

File Name: Supplementary Data 2
Description: Complete list of proteins for graphene exfoliated with 100% HS as identified by MS and analysed by MaxQuant by the method of normalised spectral counts (NSpC). Form this lists, keratin contaminations has been removed as well as proteins with NSpC < 0.1.
