# Supplementary material for: Biological recognition of graphene nanoflakes
Source: Nat Commun. 2018 Apr 20;9:1577. doi: 10.1038/s41467-018-04009-x (PMC5910434; doi:10.1038/s41467-018-04009-x)
Supplement: Supplementary file 3 — Supplementary Data 1 [file 41467_2018_4009_MOESM3_ESM.pdf]

	Protein names	Mol. weight [kDa]	SpC	NSpC %
1	Serum albumin	69.293	136	8.825879054
2	Hemoglobin subunit alpha	15.184	23	6.811614614
3	Apolipoprotein A-I	30.276	32	4.752913488
4	Hemoglobin subunit beta	15.859	14	3.969727211
5	Alpha-2-HSglycoprotein	38.418	27	3.160366427
6	Histone H4	11.367	7	2.769240074
7	Apolipoprotein E	35.979	20	2.499708315
8	Alpha-1-antiproteinase	46.103	24	2.340941079
9	Complement C3	187.25	73	1.753111188
10	Alpha-2-macroglobulin	167.57	62	1.663810449
11	Fibrinogen alpha chain	67.011	24	1.61054762
12	Transgelin-2	22.426	8	1.604155988
13	Insulin-like growth factor-binding protein	34.015	12	1.586423733
14	Kininogen-1	68.889	24	1.566642085
15	Actin, cytoplasmic 2	41.792	14	1.50641041
16	Vitamin D-binding protein	53.341	17	1.433165007
17	Histone H2B type 1-K	13.875	4	1.296389268
18	Alpha-fetoprotein	68.587	19	1.245719381
19	Inter-alpha-trypsin inhibitor heavy chain	101.51	28	1.240388215
20	Plasminogen	91.215	25	1.232486508
21	Ubiquitin-60S ribosomal protein L40	14.728	4	1.22130643
22	Histone H3.3	15.328	4	1.17349955
23	Secreted phosphoprotein 24	23.134	6	1.166296431
24	Fetuin-B	42.663	11	1.159443851
25	Glyceraldehyde-3-phosphate dehydrogenase	35.868	9	1.128349851

26	Hemoglobin subunit beta	15.954	4	1.127453999
27	Antithrombin-III	52.347	13	1.116760341
28	Tetranectin	22.144	5	1.015365398
29	Plasma serine protease inhibitor	45.296	10	0.992769841
30	Apolipoprotein C-IV	14.437	3	0.934442808
31	Junction plakoglobin	81.82	16	0.879364512
32	RaSrelated protein Rap-1	20.825	4	0.863740749
33	Adiponectin	26.133	5	0.860377736
34	Peptidoglycan recognition protein 1	21.063	4	0.853980966
35	Leukocyte cell-derived chemotaxin-2	16.32	3	0.826626889
36	Alpha-2-antiplasmin	54.71	10	0.821943022
37	Cathelicidin-4	16.479	3	0.81865106
38	Calpain small subunit 1	27.931	5	0.80499271
39	Heat shock protein beta-1	22.393	4	0.803259996
40	Pigment epithelium-derived factor	46.228	8	0.778203733
41	Serpin A3-2	46.236	8	0.778069085
42	L-lactate dehydrogenase A chain	36.597	6	0.737249
43	Carboxypeptidase	48.821	8	0.736871473
44	Protein AMBP	39.234	6	0.687696937
45	Histone H2A.V	13.509	2	0.665756203
46	Ribonuclease 4	13.741	2	0.654515723
47	Heat shock cognate 71 kDa protein	71.24	10	0.631225474
48	Clusterin O	51.113	7	0.615850213
49	Peroxiredoxin-1	22.209	3	0.607436212
50	Profilin-1	15.057	2	0.597310258
51	Prothrombin O	70.505	9	0.574025281
52	Transthyretin	15.727	2	0.571863709

53	TP-binding nuclear protein Ran	24.423	3	0.55237075
54	Apolipoprotein A-IV	43.017	5	0.522682925
55	Nucleoside diphosphate kinase A 2	17.298	2	0.519927191
56	Hemopexin	52.209	6	0.51679024
57	Arginase-1 O	35.009	4	0.513793627
58	Complement factor H	140.37	16	0.512571093
59	Cathelicidin-6	17.852	2	0.503792323
60	14-3-3 protein sigma	27.849	3	0.484417782
61	Beta-2-glycoprotein 1	38.252	4	0.470234265
62	Elongation factor 1-alpha 1	50.14	5	0.448429425
63	Peroxiredoxin-2	21.946	2	0.409810469
64	Inter-alpha-trypsin inhibitor heavy chain H3	99.55	9	0.406545982
65	60S acidic ribosomal protein P2	11.702	1	0.384280488
66	Cytochrome c	11.703	1	0.384247652
67	Dynein light chain 4, axonemal	12.009	1	0.37445668
68	High mobility group protein B2	24.033	2	0.374222966
69	Tubulin beta-4B chain	49.83	4	0.360975338
70	Adenylyl cyclase-associated protein 1	51.272	4	0.350823083
71	Annexin A2	38.612	3	0.349387517
72	Complement C1q subcomponent subunit A	25.802	2	0.348566024
73	Factor XIIa inhibitor	51.723	4	0.347764072
74	Coagulation factor V	248.98	19	0.343160717
75	Triosephosphate isomerase	26.689	2	0.336981548
76	Alpha-1B-glycoprotein	53.553	4	0.335880363
77	Beta-2-microglobulin	13.677	1	0.328789228
78	14-3-3 protein zeta/delta	27.745	2	0.324155723

79	Complement factor D	27.878	2	0.322609246
80	Insulin-like growth factor-binding protein 4	27.89	2	0.322470439
81	Plasma kallikrein	70.993	5	0.316710822
82	Fibronectin	272.15	19	0.313945086
83	Histidine-rich glycoprotein (Fragments)	44.47	3	0.30336296
84	Fatty acid-binding protein, epidermal	15.074	1	0.298318315
85	Protein-lysine 6-oxidase	47.004	3	0.28700857
86	Adenosylhomocysteinase	47.637	3	0.283194803
87	Plakophilin-1	80.179	5	0.28042569
88	Cystatin-C	16.265	1	0.276474041
89	Fibrinogen gamma-B chain	50.243	3	0.268506077
90	Lysozyme C, milk isozyme	16.782	1	0.267956756
91	Calmodulin	16.837	1	0.267081444
92	Peptidyl-prolyl ciStrans isomerase A	17.869	1	0.251656515
93	Cofilin-1	18.518	1	0.242836714
94	Lumican O	38.756	2	0.232059566
95	ADP-ribosylation factor 3	20.601	1	0.218283106
96	Gamma-glutamylcyclotransferase	21.177	1	0.212345954
97	Histone H1.2	21.355	1	0.21057599
98	RaSrelated C3 botulinum toxin substrate 2	21.424	1	0.209897791
99	Phospholipid hydroperoxide glutathione peroxidase, mitochondrial	22.24	1	0.202196505
100	Secernin-1	46.201	2	0.19466463
101	Serpin H1	46.506	2	0.193387962
102	Alpha-enolase	47.326	2	0.1900372

103	Insulin-like growth factor-binding protein 6	24.967	1	0.180111758
104	Inter-alpha-trypsin inhibitor	101.24	4	0.177670892
105	Glutathione peroxidase 3	25.663	1	0.175226991
106	Coagulation factor IX	52.045	2	0.172806236
107	Cartilage oligomeric matrix protein	82.362	3	0.163795814
108	Outer dense fiber protein	29.435	1	0.152772219
109	Voltage-dependent anion-selective channel protein 3	30.738	1	0.146296124
110	Voltage-dependent anion-selective channel protein 2	31.619	1	0.142219876
111	Elongation factor 2	95.367	3	0.141459318
112	Cadherin-1	97.936	3	0.13774864
113	Calponin-2	33.437	1	0.134487253
114	Thrombospondin-4	105.97	3	0.127305377
115	Malate dehydrogenase, mitochondrial	35.668	1	0.126075201
116	Heterogeneous nuclear ribonucleoproteins A2/B1	36.006	1	0.124891692
117	L-lactate dehydrogenase B chain	36.723	1	0.122453238
118	Hepatocyte growth factor-like protein	79.972	2	0.112460618
119	Vasodilator-stimulated phosphoprotein	40.463	1	0.111134871
120	Heat shock protein HSP 90-beta	83.252	2	0.108029844
121	Actin, alpha cardiac muscle 1	42.019	1	0.10701945

Supplementary Data 1: complete list of proteins for graphene exfoliated with 100% FBS as identified by MS and analysed by MaxQuant by the method of normalised spectral counts (NSpC). Form this lists, keratin contaminations has been removed as well as proteins with NSpC < 0.1.
