# Supplementary material for: Biological recognition of graphene nanoflakes
Source: Nat Commun. 2018 Apr 20;9:1577. doi: 10.1038/s41467-018-04009-x (PMC5910434; doi:10.1038/s41467-018-04009-x)
Supplement: Supplementary file 4 — Supplementary Data 2 [file 41467_2018_4009_MOESM4_ESM.pdf]

	Protein names	Mol. weight [kDa]	SpC	NSpC %
1	Serum albumin	69.366	140	10.02047216
2	Apolipoprotein A-I	30.777	53	8.54980849
3	Apolipoprotein E	36.154	32	4.394408504
4	Vitronectin	54.305	47	4.296994935
5	Alpha-1-antitrypsin	46.736	32	3.399423251
6	Apolipoprotein A-IV	45.398	29	3.171524563
7	Hemoglobin subunit beta	15.998	10	3.103423964
8	Apolipoprotein A-II	5.8767	3	2.534513073
9	Complement C3	187.15	91	2.414117269
10	L-lactate dehydrogenase A chain	4.5344	2	2.189863117
11	Antithrombin-III	52.602	22	2.076477482
12	Histidine-rich glycoprotein	59.578	23	1.916676057
13	Apolipoprotein C-I	8.647	3	1.722513354
14	Clusterin	48.803	16	1.627722118
15	Apolipoprotein C-III	10.852	3	1.372518704
16	Ig lambda-3 chain C regions	11.237	3	1.325493724
17	Hemoglobin subunit alpha	15.257	4	1.301660263
18	Ig kappa chain V-III region	11.746	3	1.26805491
19	Alpha-1-antichymotrypsin	47.65	12	1.250331414
20	Immunoglobulin J chain	8.1675	2	1.215759451
21	Protein IGHV3-74	12.839	3	1.160103822
22	Gelsolin	85.696	20	1.158713979
23	Inter-alpha-trypsin inhibitor heavy chain H2	105.21	24	1.132559489
24	Serum amyloid A-4 protein	14.746	3	1.010075476
25	Alpha-2-macroglobulin	163.29	32	0.97296494
26	Alpha-enolase;Beta-enolase;Gamma-enolase	36.928	7	0.941128781
27	Kininogen-1	47.883	9	0.93318545

28	Platelet factor 4	10.845	2	0.915603072
29	Ig kappa chain V-II region	11.043	2	0.899186391
30	Dermcidin	11.284	2	0.879981861
31	Ig heavy chain V-III region	11.612	2	0.855125329
32	Actin, cytoplasmic 2	41.792	7	0.83159465
33	Immunoglobulin lambda-like polypeptide 5	24.83	4	0.799815974
34	Serum amyloid P-component	25.387	4	0.782267721
35	Inter-alpha-trypsin inhibitor heavy chain H4	103.36	15	0.720519204
36	Histone H2A	6.8918	1	0.720400717
37	Cystatin-A	7.0861	1	0.700647417
38	Apolipoprotein C-IV	14.553	2	0.682313978
39	Ig alpha-1 chain C region	37.654	5	0.659273604
40	Histone H3.1	15.404	2	0.644619275
41	Pigment epithelium-derived factor	46.312	6	0.64322737
42	Inter-alpha-trypsin inhibitor heavy chain H1	101.39	13	0.636582992
43	Elongation factor 1-alpha 1	15.936	2	0.623099606
44	Apolipoprotein C-II	8.1463	1	0.609461677
45	Heparin cofactor 2	57.07	7	0.608971502
46	Fibronectin	239.62	29	0.600871681
47	Hemopexin	51.676	6	0.57645998
48	Aldo-keto reductase family 1 member C3	17.563	2	0.565376947
49	Glyceraldehyde-3-phosphate dehydrogenase	36.053	4	0.550839892
50	Haptoglobin	45.205	5	0.549149171
51	Alpha-2-HSglycoprotein	39.324	4	0.505020614
52	Plasma protease C1 inhibitor	49.757	5	0.498910471
53	Apolipoprotein D	21.275	2	0.466731625
54	Peroxiredoxin-2	10.676	1	0.465048488

55	Ig kappa chain V-III region NG9	10.729	1	0.462751203
56	Protein IGHV3-72	11.167	1	0.444600847
57	Aldo-keto reductase family 1 member C2	22.631	2	0.438766087
58	Histone H4	11.367	1	0.436778188
59	Lysozyme C	11.488	1	0.432177721
60	Ig kappa chain V-I	11.788	1	0.421178967
61	Ig kappa chain V-I	11.842	1	0.419258373
62	Ig lambda chain V-III	11.935	1	0.415991425
63	Complement C1q subcomponent subunit B	24.117	2	0.41173095
64	HCG2041221	12.145	1	0.40879849
65	Complement factor H	139.09	11	0.392648172
66	Ig kappa chain C region	25.6	2	0.387879505
67	Serotransferrin	77.063	6	0.386555752
68	Apolipoprotein B-100	515.6	39	0.375541987
69	Protein S100-A9	13.242	1	0.374932613
70	Ig kappa chain V-IV region	13.38	1	0.371065595
71	Platelet basic protein	13.894	1	0.357338251
72	Complement factor B	85.532	6	0.348280713
73	Insulin-like growth factor-binding protein 3	14.9	1	0.333211923
74	Plasminogen	90.568	6	0.328914693
75	Transthyretin	15.12	1	0.328363602
76	Plasma serine protease inhibitor	45.674	3	0.326106165
77	Erythrocyte band 7 integral membrane protein	31.73	2	0.312944069
78	Transketolase	16.559	1	0.299828351
79	C4b-binding protein alpha chain	67.033	4	0.296263492
80	Eukaryotic translation initiation factor 5A-1	16.773	1	0.296002961
81	Vitamin D-binding protein	53.02	3	0.28092367

82	Tetranectin	17.794	1	0.279018639
83	Ig gamma-2 chain C region	35.9	2	0.276593741
84	Ig gamma-4 chain C region	35.94	2	0.276285902
85	Apolipoprotein L1	18.108	1	0.274180343
86	Retinal dehydrogenase 1	54.861	3	0.271496564
87	Leucine-rich alpha-2-glycoprotein	38.177	2	0.260096794
88	Glucose-6-phosphate 1-dehydrogenase	59.256	3	0.251359744
89	Proteoglycan 4	102.51	5	0.242164553
90	Complement component C9	63.173	3	0.235774349
91	Prothrombin	70.036	3	0.212670241
92	Vimentin	49.653	2	0.199982183
93	Glutathione peroxidase 3	25.402	1	0.195451447
94	Complement C1q subcomponent subunit C	25.773	1	0.192637941
95	Myosin-V	26.456	1	0.187664713
96	Angiotensinogen	53.154	2	0.186810312
97	Alpha-2-antiplasmin	54.565	2	0.181979571
98	Ceruloplasmin	97.712	3	0.152433406
99	Alpha-1B-glycoprotein	33.455	1	0.148404055
100	Selenoprotein P	35.116	1	0.141384487
101	Plasma kallikrein	71.369	2	0.139132051
102	Ig alpha-2 chain C region	36.526	1	0.135926673
103	Peptidoglycan recognition protein 3	37.611	1	0.132005468
104	Complement C1s subcomponent	75.905	2	0.130817671
105	Beta-2-glycoprotein 1	38.298	1	0.129637518
106	Complement C1r subcomponent	76.613	2	0.129608752
107	Protein AMBP	38.999	1	0.127307307
108	Serum paraoxonase/arylesterase 1	39.731	1	0.12496181
109	Pyruvate kinase	40.189	1	0.123537726
110	Ig gamma-3 chain C region	41.287	1	0.120252323

Supplementary Data 2: complete list of proteins for graphene exfoliated with 100% HS as identified by MS and analysed by MaxQuant by the method of normalised spectral counts (NSpC). Form this lists, keratin contaminations has been removed as well as proteins with NSpC < 0.1.
